# Self-Assembling Peptide–Co-PPIX Complex Catalyzes Photocatalytic Hydrogen Evolution and Forms Hydrogels

**DOI:** 10.3390/molecules30081707

**Published:** 2025-04-10

**Authors:** Nicholas Ryan Halloran, Abesh Banerjee, Giovanna Ghirlanda

**Affiliations:** School of Molecular Sciences, Arizona State University, Tempe, AZ 85287, USA; nickhalloran@gmail.com (N.R.H.); abaner30@asu.edu (A.B.)

**Keywords:** artificial metalloenzymes, hydrogen evolution, cobalt porphyrin, catalysis, protein design

## Abstract

The sustainable production of carbon-free fuels such as hydrogen is an important goal in the search for alternative energy sources. Herein, we report a peptide-based system for light-driven hydrogen evolution from water under neutral conditions. The M1 peptide is an ABC triblock polymer featuring two coiled-coil alpha-helical regions flanking a water-soluble, polyanionic, intrinsically disordered region. M1 formed a hydrogel at high concentrations upon binding to cobalt protoporphyrin IX. This process is driven by the terminal regions, which coordinate the metalloporphyrin through histidine residues and form helical oligomers interconnected by flexible, intrinsically disordered regions, resulting in network formation. Co-M1 catalyzes hydrogen production upon irradiation in the presence of a photosensitizer and a sacrificial electron donor; the activity of Co-M1 is eight times higher than that of free Co-PPIX.

## 1. Introduction

The increasing demand for sustainable and environmentally friendly energy sources has spurred significant interest in hydrogen as a carbon-free alternative to fossil fuels [1]. To realize the potential of hydrogen as a large-scale energy carrier, its production must be efficient, scalable, and environmentally benign, relying on aqueous solvents, low temperatures, and Earth-abundant catalysts [2,3]. Among potential catalytic systems, cobalt complexes, particularly organometallic cobalt porphyrins, have demonstrated promising activity for hydrogen evolution under electrocatalytic and photocatalytic conditions [4,5,6,7,8,9,10]. However, these systems often suffer from limitations such as a reliance on organic solvents, synthetic complexity, and limited stability under operational conditions.

One promising strategy to address these issues employs artificial metalloenzymes, which embed metal cofactors into protein scaffolds to impart water solubility, protect the active site from degradation, and provide primary and secondary coordination spheres that can be easily remodeled using mutagenesis [11,12,13,14,15,16,17]. In recent years, our group and others have applied this approach to develop artificial enzymes for sustainable hydrogen production by swapping cobalt protoporphyrin IX for heme in heme-binding proteins such as myoglobin, cytochrome c, and cytochrome b_562_, as well as heme-binding synthetic peptides, and demonstrated improved catalytic activity compared to free Co-PPIX under photocatalytic and/or electrocatalytic conditions [18,19,20,21,22,23,24,25,26,27,28,29].

We hypothesized that embedding cobalt-based hybrid catalysts within peptide-based biomaterials could further enhance stability and catalytic robustness and expand the scope of applications [11,30,31]. Peptide-based hydrogels, in particular, represent a promising platform owing to their unique structural properties and responsiveness [32,33,34,35]. These materials undergo sol–gel transitions driven by the folding and association of coiled-coil domains, forming porous networks stabilized by intrinsically disordered regions. The sequences were modeled on a triblock polymer pattern first introduced by the Tirrell group, consisting of terminal alpha helical-forming domains derived from leucine zippers and flanking a central hydrophilic intrinsically disordered region (IDR) that swells in water at a neutral pH [32]. Concentration-dependent folding of the terminal regions into dimeric coiled-coils mediates intermolecular assembly and triggers the formation of a porous network hydrogel, while the central region maintains the water solubility of the assembly. This pattern has been further refined using leucine zippers, four- and five-helix bundles, and other coiled coils to modulate their mechanical properties as well as impart the ability to bind and release small molecules [34,35,36,37,38]. Fusion of these basic scaffolds to enzymes or other globular proteins expanded the functionality of the hydrogels [36,38,39,40].

Herein, we present a novel peptide-based triblock polymer, M1, designed to form hydrogels upon binding to Co-PPIX and catalyzing light-driven hydrogen evolution. The M1 sequence features terminal coiled-coil domains that coordinate metalloporphyrins through histidine residues and a central intrinsically disordered region (IDR) that promotes water solubility. This design enables M1 to form a hydrogel network at high concentrations, providing a stable and flexible scaffold for Co-PPIX coordination and catalytic activity. Our results demonstrate that M1-Co-PPIX not only enhances the hydrogen evolution rate but also significantly improves the thermal and oxidative stability of the catalyst. These findings highlight the potential of peptide-based biomaterials to enable functionalization with catalytic cofactors while maintaining water solubility and structural integrity under diverse conditions and pave the way for the development of versatile, genetically encoded catalytic platforms.

## 2. Results

### 2.1. Design of M1

**Figure 1:**
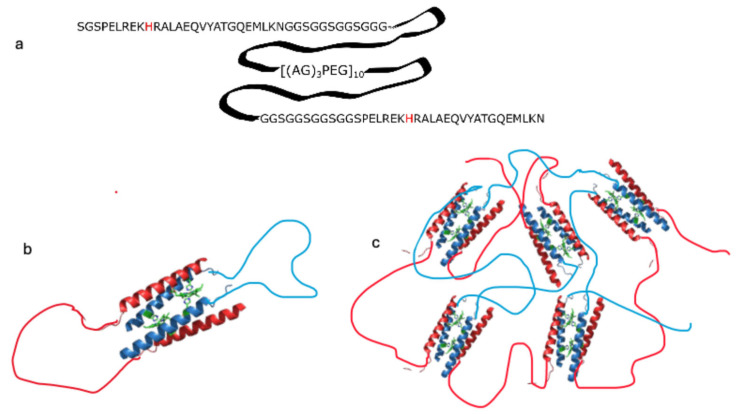
Sequence of M1, an ABC triblock peptide containing a coiled-coil sequence in blocks A and C and a disordered linker in block B (panel **a**). Proposed assembly of M1 in the presence of metal porphyrins, forming a dimer with 2:2 stoichiometry at low concentration (panel **b**; M1 monomers are shown in blue and red) and forming a network gel at high concentration (panel **c**; monomers are shown in blue and red).

The M1 sequence is a variation of the triblock polymer pattern in which the terminal coiled-coil regions fold into tetramers, while the linker region is the Ala-Gly rich sequence [(AG)_3_PEG]_10_, which is predicted to be intrinsically disordered [32,41]. The coiled-coil forming sequence at the termini (A and C blocks) corresponds to D_2_-heme, a peptide that is unfolded in the apo state and cooperatively folds into a well-folded four helix bundle upon binding to two hemes [42]. We hypothesized that a triblock sequence containing D_2_-heme would be unfolded but would form a hydrogel in the presence of heme. In M1, the D_2_ sequence serves as a multivalent “sticker” mediating self-assembly, but only when bound to heme: in the absence of heme coordination, the D_2_ block is disordered (Figure 1a). Given the antiparallel topology of the four-helix bundle formed by D_2_, M1 can form two types of assemblies: a “closed dimer” structure, in which two M1 monomers bind two heme molecules (Figure 1b), and an open multimeric structure, in which several M1 molecules assemble around the heme cofactors (Figure 1c).

### 2.2. Binding of Fe-PPIX and Co-PPIX

**Figure 2:**
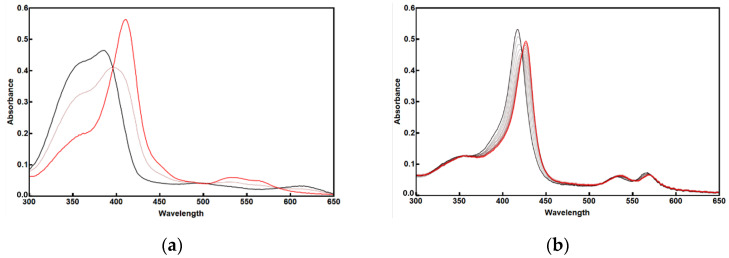
UV-Vis spectra of M1-Fe-PPIX (**a**) and M1-Co-PPIX (**b**). The free metalloporphyrins are shown in black and the complexes with M1 are shown in red; 50% mix is in light red. Conditions: 3.8 mM free metalloporphyrin in PBS, to which M1 Is added in increments to a 10 mM concentration.

We assessed the binding of M1 to Fe-PPIX, analogous to the D_2_ peptide, and its extension to Co-PPXI, using UV-vis spectroscopy. The addition of M1 to ferric heme induced a Soret band at 415 nm (ε = 76 mM^−1^ cm^−1^ per site) and typical α- and β-bands at 562 and 529 nm, respectively, which were indistinguishable from those observed for the D_2_ peptide. Similarly, M1 binds to Co-PPIX, as shown by the bathochromic shift in the Soret band of free metalloporphyrin from 415 nm to 426 nm upon the addition of M1. The spectra showed distinct isosbestic points (398 and 422 nm for Fe-PPIX and Co-PPIX, respectively), indicating two-state equilibrium between the free and bound metalloporphyrin species (Figure 2).

A binding isotherm obtained by addition of M1 to Co-PPIX indicates a 1:1 stoichiometry, consistent with the design (Supplementary Figure S1). The simplest possible folding conformation would have a 2:2 binding ratio following the self-assembly of two M1 peptides forming two 4-helix bundles in a “closed” dimer (Figure 1b). Other arrangements resulting from “open” oligomers also show an apparent 1:1 stoichiometry.

**Figure 3:**
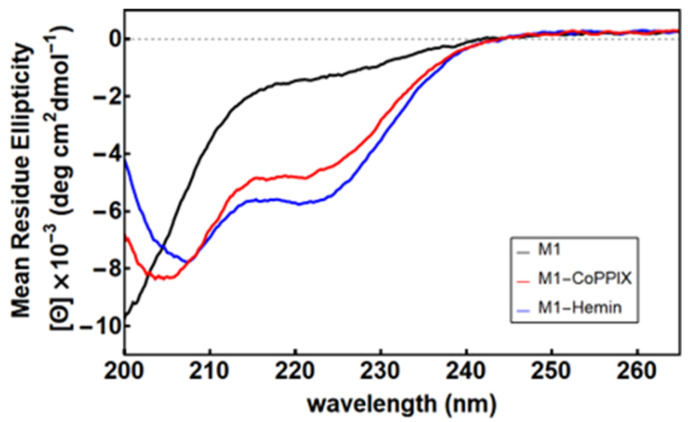
Circular dichroism spectra of M1 in the apo state (black) and with excess Co-PPIX (red) and Fe-PPIX (blue). Conditions: 15 µM [M1], 10 mM phosphate buffer (pH 7.0), and 50 µM metalloporphyrin.

To investigate the structural changes associated with metalloporphyrin binding, we compared the circular dichroism (CD) spectra of M1 in the apo-, heme-, and Co-PPIX-bound states (Figure 3). Apo M1 displayed a predominantly unfolded spectrum, while metalloporphyrin-bound M1 exhibited minima at 222 nm and 208 nm (heme) or 205 nm (Co-PPIX), indicating an increase in α-helical structure formation in the background of the M1 spectrum. Fe-PPIX-M1 underwent thermal denaturation with an apparent T_m_ of 57.6 °C, comparable to that measured for D_2_-Heme, while Co-PPIX-M1 retained high helical content at temperatures as high as 90 °C (Figure S1).

The Fe(III)- and Co(III)-bound M1 peptides exhibit distinct differences in their thermal stability and unfolding behavior. Whereas Fe(III)-M1 shows cooperative denaturation, Co(III)-M1 demonstrates greater thermal stability but noncooperative unfolding, suggesting multiple coexisting conformational states. We hypothesize that these differences may result from the distinct axial ligand binding characteristics and ligand-exchange propensities of Co(III) and Fe(III). Specifically, the lower ligand exchange rate of Co(III) likely leads to kinetically trapped intermediates, while the Fe(III) complex equilibrates more readily to a stable folded structure.

**Figure 4:**
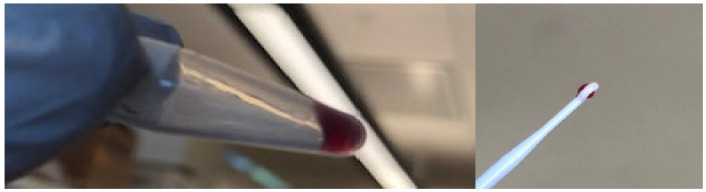
M1-Co-PPIX underwent gelation upon heating to 50 °C followed by cooling to 4 °C.

Thus, we tested whether Co-M1 could exhibit gel-sol behavior by heating solutions of Co-PPIX-M1 to 50 °C, followed by cooling to 4 °C. At protein concentrations above 4% *w*/*v* (7 mM), Co-M1 formed a hydrogel (Figure 4).

The remarkable stability against thermal denaturation displayed by Co-PPIX-M1 suggests that this material can be exploited to generate catalytically active biomaterials. “Free” Co-PPIX catalyzes hydrogen production under photocatalytic conditions; however, this application is hampered by the limited stability of “free” Co-PPIX in aqueous solutions to oxygen, heat, and hydrogen peroxide generated during photocatalysis through a combination of multiple possible pathways including dimerization, “H”- or “J”-type aggregates, or dioxygen bridges [20].

To assess whether binding to M1 protected Co-PPIX from degradation, we evaluated its stability under oxidative, thermal, and reactive conditions. Free Co-PPIX showed significant degradation after exposure to oxygen and heat (60 °C, as evidenced by a reduction in Soret band intensity). In contrast, M1-bound Co-PPIX exhibited remarkable stability under both thermal and oxidative conditions. Next, we compared the stability of the two species in the presence of H_2_O_2_. Reactive oxygen species (ROS), including H_2_O_2_, are generated under photocatalytic conditions and cause the degradation of porphyrins [25,26]. Incubation of free Co-PPIX with 1.5% H_2_O_2_ (467 mM) for an hour resulted in almost complete disappearance of the Soret band, whereas Co-PPIX-M1 retained approximately 85% of its Soret intensity (Figure S2).

### 2.3. Photocatalytic Hydrogen Evolution

We investigated whether the increased stability of M1 bound Co-PPIX translates to an increase in hydrogen evolution under photocatalytic conditions, using [Ru(bpy)_3_]^2+^ as a photosensitizer and ascorbic acid as a sacrificial electron donor. The mechanism of hydrogen evolution (HER) involves light absorption by a photosensitizer (PS) and the reduction of its excited state by a sacrificial electron donor (SD); the reduced photosensitizer (PS^−^) reduces the catalyst to its active state. Hydrogen evolution involves the formation of a metal hydride, which is protonated and releases molecular hydrogen with a total transfer of two protons and two electrons [25,43,44].

**Figure 5:**
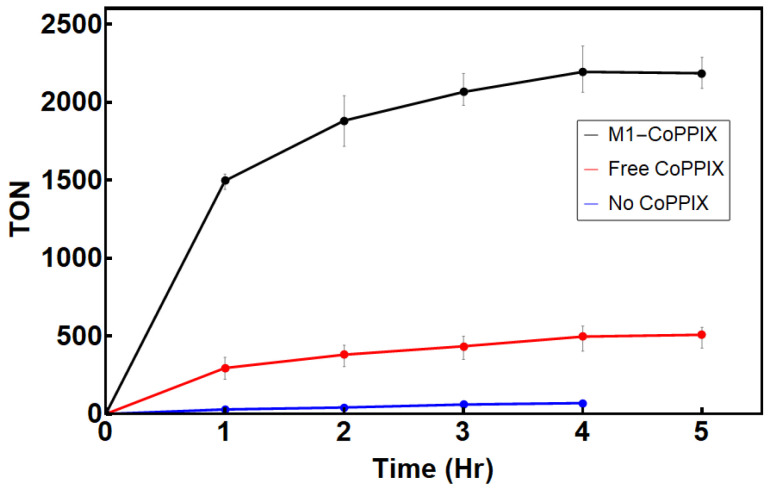
H_2_ production for black: M1-bound Co-PPIX; red: free Co-PPIX; blue: H_2_ produced from [Ru(bpy)_3_]^2+^. The reaction conditions were as follows: 1 µM Co-PPIX, 1mM [Ru(bpy)_3_]^2+^, and 200 mM ascorbic acid in 500 mM sodium phosphate buffer (pH 6.5). The reaction was performed under anaerobic conditions at 25 °C.

Hydrogen production was monitored using gas chromatography. Under anaerobic conditions, M1-Co-PPIX achieved a turnover number (TON) of 2180 after 5 h, representing a four-fold increase compared to free Co-PPIX (TON = 510; Figure 5). This enhancement was even more pronounced under aerobic conditions, with M1-Co-PPIX displaying an eight-fold increase in TON compared with free Co-PPIX (Table 1). Although a direct comparison is complicated by differences in experimental conditions, the reported TON of cobalt porphyrin-binding proteins is in the 10^2^–10^3^ range [20,21,23], while for peptide-based systems containing covalently attached cobalt porphyrin, the reported TON is in the 10^3^–10^4^ range [19,45].

**Figure 6:**
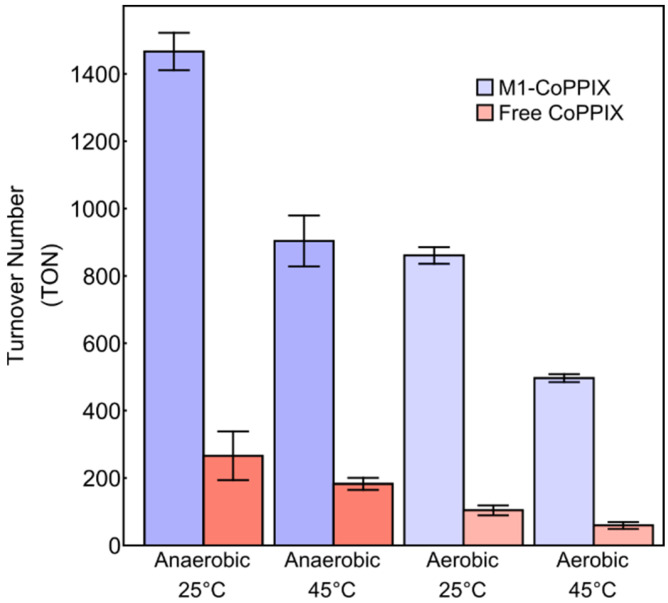
Turnover numbers after 1 h of illumination with 1 µM of catalyst as a function of temperature and air for Co-PPIX-M1 (blue) and free Co-PPIX (red) under different conditions. Conditions: 1 µM Co-PPIX, 1 mM [Ru(bpy)_3_]^2+^, and 200 mM ascorbic acid in 500 mM sodium phosphate buffer (pH 6.5).

Hydrogen evolution remained robust at elevated temperatures (45 °C) and in the presence of oxygen, with M1-Co-PPIX showing higher catalytic activity and stability than free Co-PPIX under all conditions tested (Figure 6). A comparison of the activity of Co-PPIX-M1 as a function of temperature and oxygen content is presented in Table 1, along with the enhancement factor over free Co-PPIX. As expected, the highest activity was observed under anaerobic conditions at 25 °C. Increasing the temperature from 25 °C to 45 °C under anaerobic conditions lowered the amount of molecular hydrogen produced but did not alter the enhancement factor of M1-Co-PPIX compared to free Co-PPIX. Hydrogen evolution was also reduced under aerobic conditions; however, Co-PPIX-M1 performed much better than free Co-PPIX, as shown by an enhancement factor of 8.3 at 25 °C and 8.4 at 45 °C. These results underscore the protective effect of the M1 scaffold on Co-PPIX, enhancing both its efficiency and durability under challenging conditions.

## 3. Materials and Methods

### 3.1. Protein Purification

Cells were streaked from a frozen stock onto an agar plate containing kanamycin. A single colony was selected with a 10 µL pipette tip and transferred into a 5 mL liquid culture of LB medium. The culture was incubated overnight at 37 °C until saturation and then transferred to a 2 L flask containing 1 L of LB medium. The culture growth was monitored at OD600 until an optical density of 0.6. Protein expression was induced by adding 1 mM IPTG, followed by incubation at 37 °C for 4 h. Cells were harvested by means of centrifugation at 5000× *g* for 30 min, frozen, and stored at −20 °C until purification.

The frozen cell pellet was resuspended in 30 mL of binding buffer (20 mM sodium phosphate, 500 mM NaCl, 20 mM imidazole, pH 7.5) and lysed by sonication on ice in 30 s intervals repeated 12 times. After sonication, the cell debris was removed by means of centrifugation at 5000× *g* for 30 min. The supernatant, containing the His-tagged protein, was passed through a 5 mL HisTrap HP (Cytiva, Marlborough, MA, USA) column using a peristaltic pump. The resin was washed with 50 mL of binding buffer to remove non-specifically bound proteins, followed by elution with 20 mM sodium phosphate, 500 mM NaCl, and 500 mM imidazole (pH 8.0). The protein-containing fractions were desalted on a PD-10 desalting column with 20 mM sodium phosphate buffer (pH 7.5). The His-tag was removed via overnight digestion with TEV protease in 1 mM DTT and 0.5 mM EDTA at 4 °C followed by purification in a HisTrap HP column. The flow-through containing the M1 protein was concentrated and further purified by RP-HPLC using a semi-preparatory Jupiter^®^ C18 column (250 mm × 4.6 mm, Phenomenex, Terrance, CA, USA). A linear gradient of 1%/min from 100% solvent A (0.1% TFA in water) to 100% solvent B (95% acetonitrile, 0.1% TFA, and 4.9% water) was applied. A single peak was collected and lyophilized to yield pure cleaved protein, as confirmed by MALDI-TOF mass spectrometry. The total crude protein yield ranged from 20 to 30 mg/L of culture, with purified yields of 15 to 20 mg/L.

### 3.2. Binding Assay

Binding to Co-PPIX was assessed in an anaerobic chamber (Coy) by adding aliquots of protein to a solution of Co-PPIX in a 1 cm quartz cuvette, and UV-Vis spectra were collected at each point using an Ocean Optics USB4000 spectrophotometer (Ocean Optics, Dunedin, FL, USA).

### 3.3. Circular Dichroism (CD)

CD spectra were recorded on a JASCO J-815 (JASCO, Piscataway, NJ, USA) spectropolarimeter with scans from 280 nm to 200 nm at 50 nm/s and a data integration time of 4 s at 5 °C; three consecutive scans were averaged. Co-PPIX-M1 and Fe-PPIX-M1 were prepared by adding free porphyrin from a stock solution in 1 M KOH to final concentrations of 15 µM M1 and 50 µM porphyrin in 10 mM sodium phosphate buffer (pH 7.0). Thermal denaturation was monitored at 222 nm as the temperature increased from 5 °C to 90 °C at a rate of 0.3 °C/min.

### 3.4. Stability Tests

UV-Vis spectroscopy was used to assess the solubility and stability of Co-PPIX under various conditions. Oxygen and heat tolerances were tested by preparing a 1 mL of Co-PPIX solution at 3 µM (ε417 = 143,500 M^−1^ cm^−1^, determined by ICP-MS). Aliquots (400 µL) were transferred to Eppendorf tubes, to which 10 µL of either the buffer or M1 protein was added. The tubes were kept in the dark at room temperature for 1 h and then heated to 60 °C for 1 h before measuring the UV-Vis spectra.

The stability of Co-PPIX against H_2_O_2_ was also tested by comparing the UV-Vis spectra before and after the addition of 10 µL of 30% H_2_O_2_ to a final concentration of 467 mM. The samples were kept in the dark at room temperature for 1 h and the spectra were measured again.

### 3.5. Hydrogen Evolution

The Co-PPIX stock solution was prepared by dissolving the solid in dimethyl sulfoxide (DMSO) at a concentration of 1 mg/100 µL and then diluting 1:1000 in 500 mM sodium phosphate buffer (pH 6.9). The concentration was verified by UV-Vis. Co-PPIX was added to a fivefold excess of the M1 protein and incubated at room temperature for 30 min. Tris(bipyridine)ruthenium(II) chloride ([Ru(bpy)_3_]^2+^) was dissolved in 500 mM sodium phosphate buffer (pH 6.9) as a photosensitizer and ascorbic acid was dissolved as an electron donor. Reactions were prepared in Eppendorf tubes with final concentrations of 5 µM M1, 1 µM Co-PPIX, 200 mM ascorbic acid, and 1 mM [Ru(bpy)_3_]^2+^ in 500 mM sodium phosphate buffer (pH 6.5), transferred to 2 mL glass vials, sealed, and purged with nitrogen gas. Samples were placed in a HepatoChem Temperature-Controlled PhotoRedOx Box (HepatoChem, Beverly, NJ, USA), kept at 25 °C through water circulation, and illuminated with an EvoluChem 55 mW/cm^2^ blue LED (Advion InterChim Scientific, Ithaca, NY, USA).

Hydrogen evolved from the reactions was quantified using a gas chromatograph (SRI Instruments, Torrance, CA, USA) equipped with a thermal conductivity detector (TCD) and a 3′ × 1/8″ molecular sieve 5 Å column. Argon was employed as the carrier gas and the TCD temperature was maintained at 100 °C to facilitate the detection of hydrogen, oxygen, and nitrogen gases in the samples. At one-hour intervals, the reaction vials were removed from the light source, inverted, and allowed to stand on the bench for 15 min. A 250 µL Hamilton gas-tight syringe (Hamilton Company, Reno, NV, USA), purged with argon, was used to inject 100 µL of argon into each inverted vial. Immediately following this, 100 µL of headspace gas was withdrawn and injected into a gas chromatography column. This process was repeated for all the samples before they were returned to the photoreactor. All GC experiments were performed in triplicate, unless otherwise specified.

Turnover numbers were determined by calculating the total moles of H_2_ produced per mole of catalyst:

$$Turnover\;Number=\frac{Moles\;H_{2}}{Moles\;Co-PPIX}$$

The total amount of H₂ produced was calculated using a standard curve (Supplementary Materials).

## 4. Conclusions

In this study, we showed that the addition of metalloporphyrins to an ABC triblock peptide containing coiled-coil forming motifs separated by a disordered spacer results in two distinct concentration-dependent behaviors. At low concentrations, binding of metalloporphyrins results in the formation of α-helical structures with an apparent 1:1 stoichiometry, which is stable against thermal denaturation and protects the cofactor from chemical degradation. At high concentrations, binding results in the formation of hydrogels that remain stable at room temperature for several months. We investigated the catalytic activity of M1-bound cobalt metalloporphyrins for proton reduction under photocatalytic conditions. We found that the intrinsic hydrogen evolution activity of Co-PPIX was greatly increased in Co-PPIX-M1, as shown by a 5-fold increase in anaerobic turnover and an 8-fold increase in aerobic turnover. Furthermore, Co-PPIX-M1 was stable at high temperatures and under oxidative conditions, protecting the cofactor from degradation.

While this study demonstrates catalytic activity primarily in solutions, the formation of a stable hydrogel at higher concentrations suggests intriguing potential applications, such as immobilization on electrodes or other surfaces. The catalytic performance of Co-M1 in the hydrogel state has not yet been explored and represents an important direction for future studies. Due to poor light permeability potentially interfering with photocatalytic conditions, these biomaterials might be applied more readily under electrocatalytic conditions, as the incorporated metalloporphyrin could serve a dual purpose in conducting electrons from the electrode and in catalyzing reactions [33,38,46,47]. Previously, these two functionalities were split in peptide-based block polymers containing a metal complex and an enzyme, laccase, that catalyzes the reduction of dioxygen to water at a neutral pH when absorbed on glassy carbon electrodes [38]. To this end, the gelation point for M1 could be optimized using established methods, such as the addition of disulfide bridges, the redesign of the linker sequence, or longer coiled-coil forming sequences [32,48,49,50,51]. Furthermore, exchanging Co-PPIX with other metalloporphyrins would give access to a much wider range of reactions.

## Figures and Tables

**Table 1:** Activity data as a function of temperature and oxygen concentration after 1 h irradiation.

Atmosphere	Temperature °C	Free Co-PPIX TON	M1-Co-PPIX TON	Enhancement Factor
Anaerobic	25	266	1466	5.5
	45	182	904	5.0
Aerobic	25	104	861	8.3
	45	59	496	8.4

## Data Availability

Dataset available on request from the authors.

## Supplementary Materials

The following supporting information can be downloaded at: https://www.mdpi.com/article/10.3390/molecules30081707/s1, Figure S1. Titration of CoPPIX (3.8 μM) with M1 peptide. The binding isotherm could not be analyzed with a 1:1 binding model (solid line), despite an apparent 1:1 stoichiometry; Figure S2. Top: CD-monitored thermal denaturation of Fe-M1 (left) and Co-M1 (right). Bottom:UV-Vis spectra of free Co-PPIX (left) and Co-M1 (right) after incubation at 25 and 60 C.; Figure S3: Right: UV-Vis spectra of free CoPPIX (solid line) and CoPPIX + 1.5% H_2_O_2_ after 1 h (dashed line). Left: UV-Vis spectra of Co-M1(solid)line and Co-M plus 1.5% H_2_O_2_ after 1 h (dashed line); Figure S4. Standard curve for the GC analysis of H_2_.
