# Supplementary material for: Self-Assembling Peptide–Co-PPIX Complex Catalyzes Photocatalytic Hydrogen Evolution and Forms Hydrogels
Source: Molecules. 2025 Apr 10;30(8):1707. doi: 10.3390/molecules30081707 (PMC12029171; doi:10.3390/molecules30081707)
Supplement: Supplementary file 1 [file molecules-30-01707-s001.zip › molecules-3469408-supplementary.pdf]

Supplementary Material

1- Binding of Co-PPIX to M1.

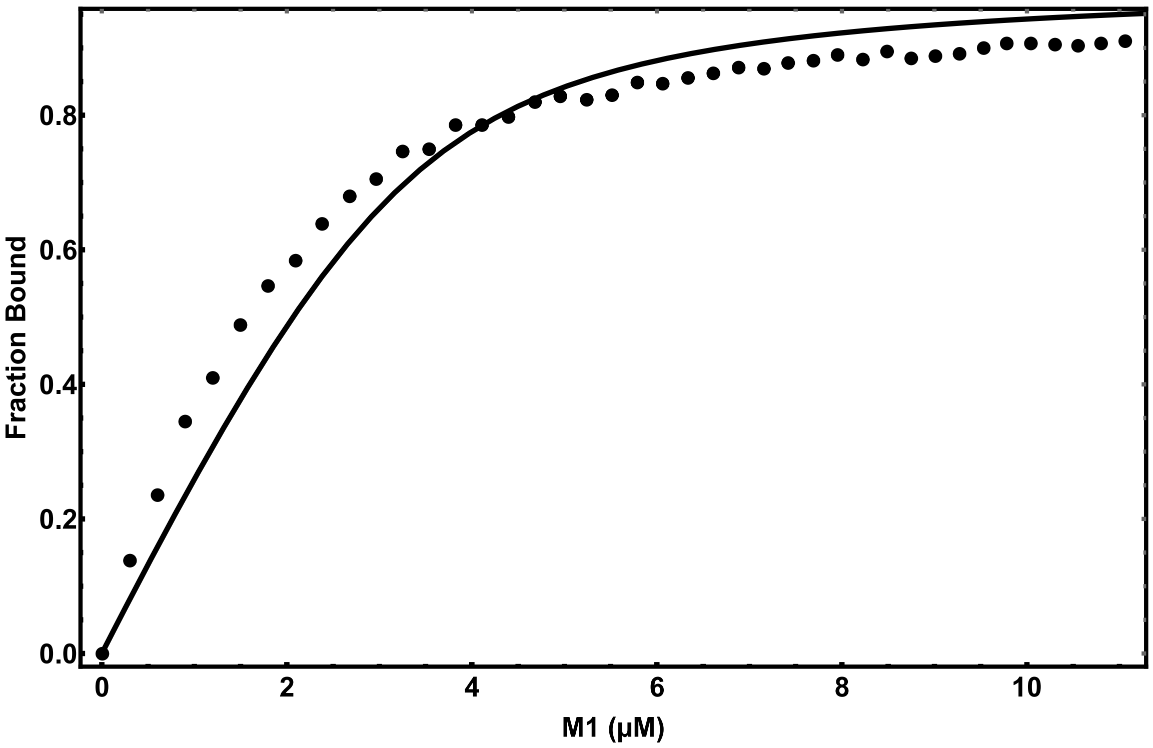

Figure S1. Titration of CoPPIX (3.8 µM) with M1 peptide. The binding isotherm could not be analyzed with a 1:1 binding model (solid line), despite an apparent 1:1 stoichiometry.

2- Stability to heat and hydrogen peroxide

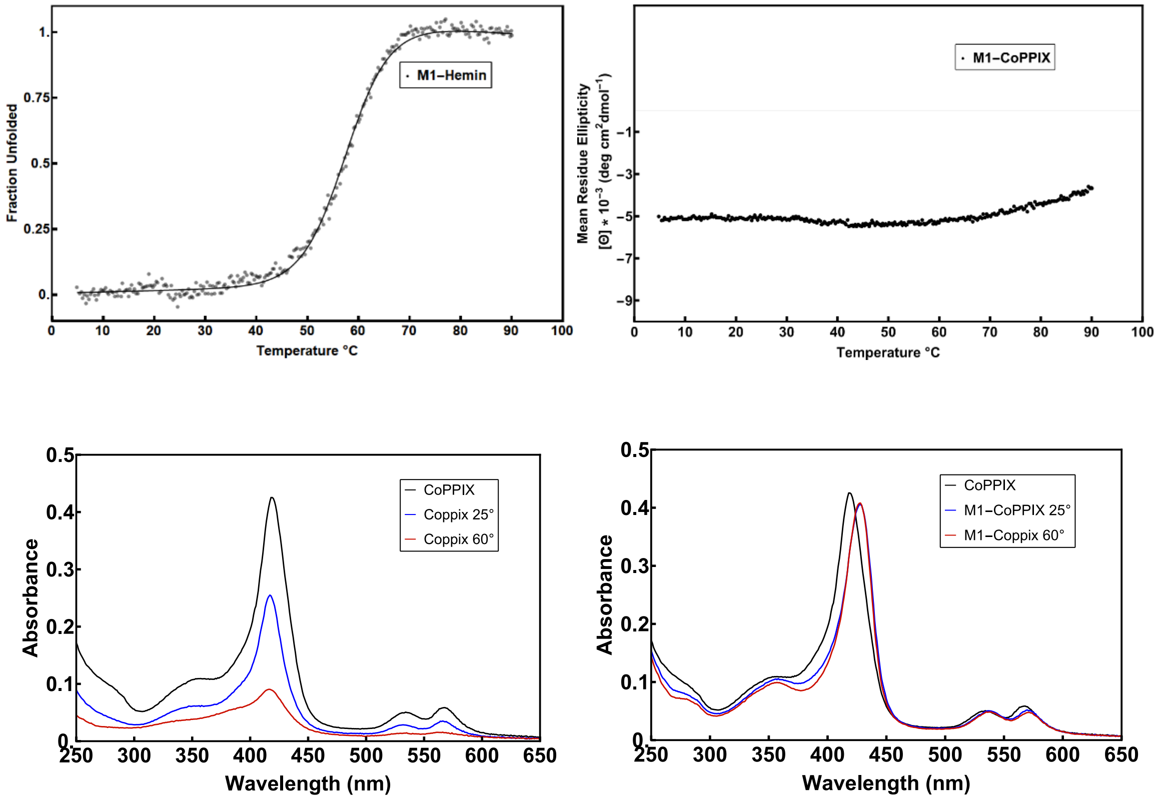

Figure S2. Top: CD-monitored thermal denaturation of Fe-M1 (left) and Co-M1 (right). Bottom:UV-Vis spectra of free Co-PPIX (left) and Co-M1 (right) after incubation at 25 and 60 C.

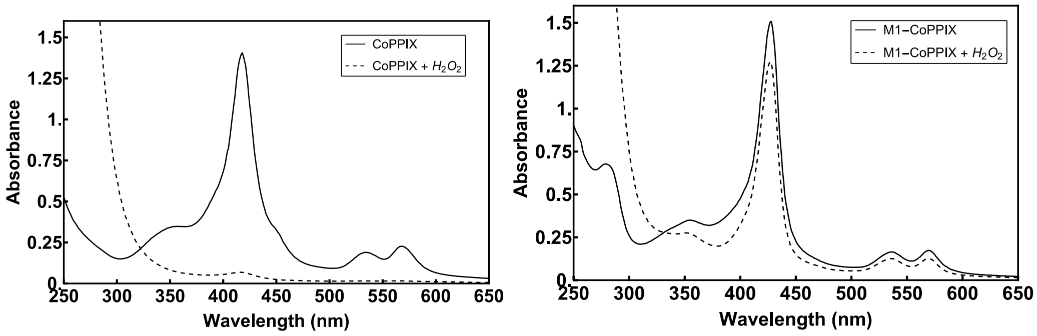

Figure S3 Right: UV-Vis spectra of free CoPPIX (solid line) and CoPPIX + 1.5% H_2O_2 after 1 h (dashed line). Left: UV-Vis spectra of Co-M1(solid)line and Co-M plus 1.5% H_2O_2 after 1 h (dashed line).

3- Hydrogen production- Standardization curve

A standard curve for H_2 was plotted to calculate the amount of H_2 produced from the area under the curve determined using gas chromatography. Known moles of H_2 were injected into the GC column in triplicate and then plotted against the average area of the peak. Linear regression gave the line shown below, with an R^2 value of 0.9986. The unknown amounts of H_2 were calculated by substituting the area under the GC peak for "x" in the formula below.

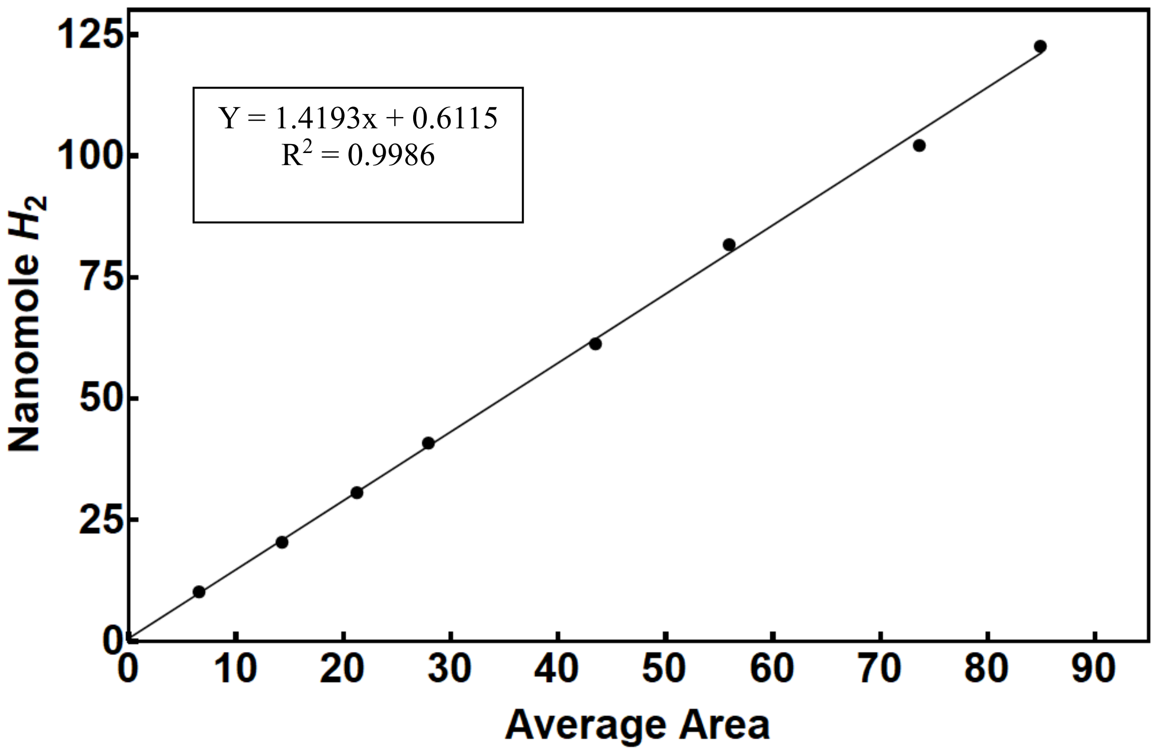

Figure S4. Standard curve for the GC analysis of H_2.
